# Whole exome sequencing of human papillomavirus-related multiphenotypic sinonasal carcinoma: a case report

**DOI:** 10.3389/fonc.2024.1448213

**Published:** 2024-09-10

**Authors:** María Camila Cubides-Córdoba, Paula Sánchez-Fernández, Guillermo E. Mendoza-Pacas, Virginia N. Cabal, Rocío García-Marín, Sara Lucila Lorenzo-Guerra, Fabián García-Velasco, Mario A. Hermsen, José Luis Llorente

**Affiliations:** ^1^ Department of Otorhinolaryngology and Head and Neck Surgery, Central University Hospital of Asturias, Oviedo, Spain; ^2^ Department of Pathological Anatomy, Central University Hospital of Asturias, Oviedo, Spain; ^3^ Department of Head and Neck Cancer, Health Research Institute of the Principality of Asturias, Oviedo, Spain; ^4^ Department of Otorhinolaryngology and Head and Neck Surgery, Son Llàtzer University Hospital, Mallorca, Spain

**Keywords:** sinonasal cancer, HPV-related multiphenotypic carcinoma, basaloid tumor, whole exome sequencing, case report

## Abstract

Human Papillomavirus (HPV) related Multiphenotypic Sinonasal Carcinoma (HMSC) is a rare tumor with features of both atypical squamous cell and adenoid cystic carcinoma, making diagnosis challenging. Approximately 80% of HMSC cases carries HPV type 33 followed by type 35. We present a patient with HMSC. Pathological classification was aided by immunohistochemistry (IHC). The presence of HPV-DNA was tested using PCR and HPV E6/E7 expression by RNA *in situ* hybridization (RNA ISH). Whole exome sequencing (WES) was used to identify somatic gene mutations and copy number alterations. A 55-year-old male presented with an HMSC in the right nostril. Histological examination showed a solid basaloid subtype with mucinous spaces and ductal structures. IHC showed positive staining for SOX-10, SMA, p40, p63, PanCK, CK8 and MYB. Diffuse positive staining for p16 was observed and PCR and RNA ISH indicated the presence of HPV type 35. The patient was treated with endoscopic surgery and radiotherapy and is currently alive and recurrence-free after 16 months of follow-up. WES revealed 38 somatic sequence variants and several chromosomal regions with copy number alterations, including a copy number gain at 6q23 where *MYB* is located. *EP300, ZNF22, ZNF609* and *LRIG3* are some of the genes whose mutations were indicated as probably pathogenic. We did not find mutations predictive for drug response according to the ESMO Scale for Clinical Actionability of Molecular Targets database. This is the first report of WES analysis of an HMSC, in this case associated with HPV type 35. The detected mutation in *EP300* and the overexpression of *MYB* may serve as molecular targets for personalized therapy.

## Introduction

Human Papillomavirus-Related Multiphenotypic Sinonasal Carcinoma (HMSC) was first described by Bishop et al. (1) in 2013 as a sinonasal HPV-related carcinoma with adenoid cystic carcinoma-like features. Subsequently, it was discovered that it had histological characteristics similar not only to adenoid cystic carcinoma but also to epithelial myoepithelial carcinoma and basal cell adenocarcinoma (2). This tumor entity was renamed Human papillomavirus (HPV)-related multiphenotypic sinonasal carcinoma (HMSCs) in 2017 (2) and officially recognized by the WHO in 2022 (5^th^ edition) (3).

HMSC exhibits the morphological features of a salivary gland tumor (mixed ductal and myoepithelial elements), superficial squamous epithelial dysplasia, and, to a lesser extent, sarcomatous transformation, cartilaginous differentiation, cellular anaplasia, and hemangiopericytoma-like vasculature, partly explaining its characterization as a “multiphenotypic” malignancy (2, 4, 5). HMSC is diffusely positive for p16, cytokeratin, SOX-10, and myoepithelial markers S-100 and SMA (2, 5, 6). A feature is the presence of high-risk HPV, most commonly type 33 followed by other such as 35, 16, 56 and 52. The t(6;9) MYB-NFIB rearrangement that characterizes adenoid cystic carcinoma is absent (2, 4, 7–9) but cMYB expression is variable in HMSC (10, 11).

It is a rare tumor, whose estimated prevalence is unknown because few cases have been described in the literature. Patients diagnosed with HMSC were clinically characterized by being young at diagnosis (50-60 years), without any sex predisposition (7). Most cases originate in the nasal cavity and are diagnosed in the initial stages (12, 13). Unlike other sinonasal carcinomas, the prognosis is considered relatively good despite its high-grade histopathological appearance (necrosis, active mitotic activity, and cellular anaplasia) (2, 14, 15). Because of this reason, it is important to distinguish HMSC from other, more aggressive sinonasal carcinoma. Local recurrence rates are high, between 24% and 36%, but regional and distant metastases are extraordinarily rare (5, 13). Therefore, it has been argued that chemotherapy does not have an important role in the treatment of HMSC (16). Treatment consists of surgical resection with or without adjuvant radiation, although the adjuvant benefit of the latter is unclear (13).

We present clinical, histopathological, and genetic details of a patient with HMSC surgically treated by endoscopic approach and adjuvant radiotherapy.

## Case description

**Figure 1:**
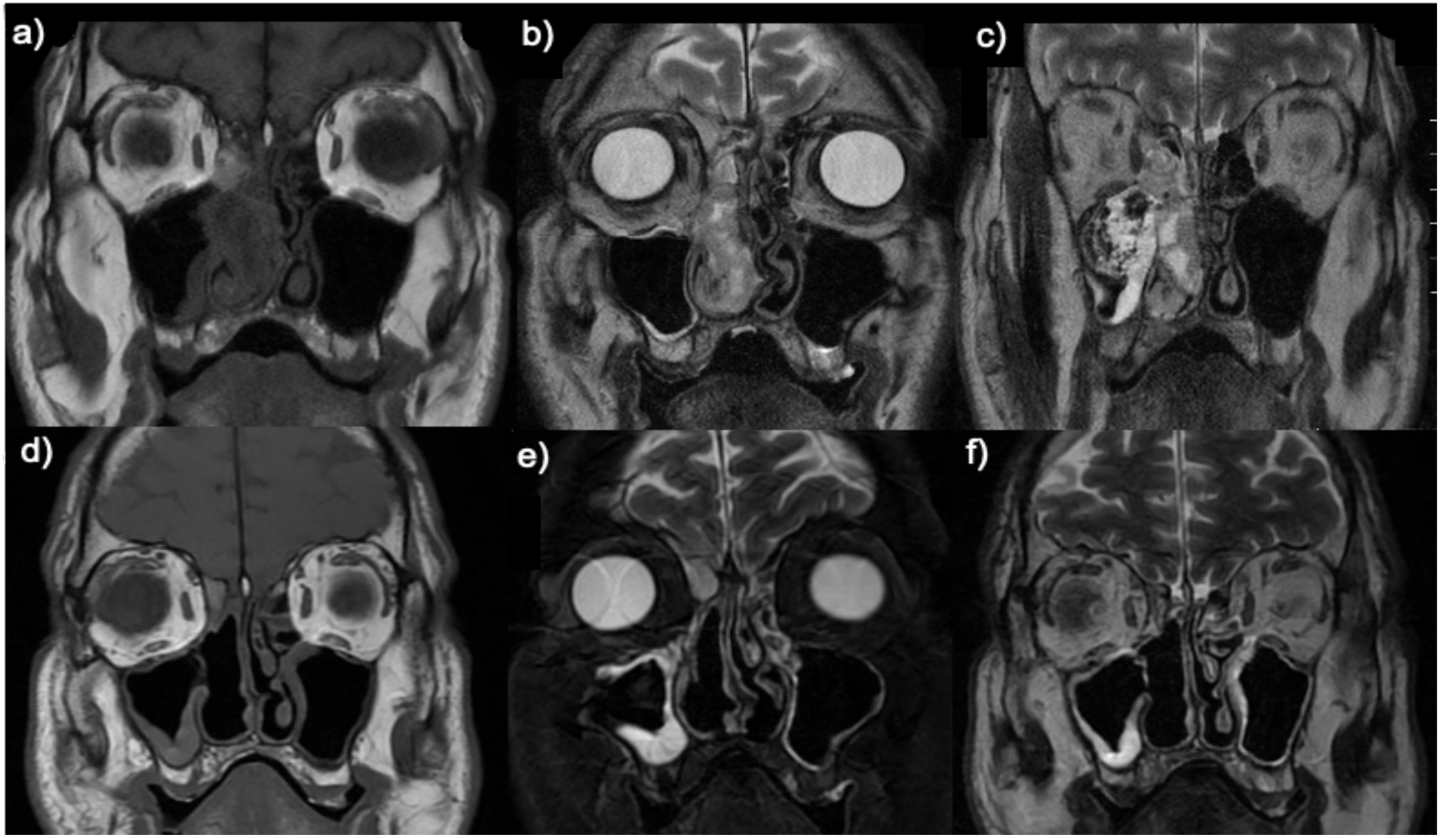
Preoperative **(A–C)** and postoperative **(D–F)** coronal T1 **(A, D)** and T2 **(B, C, E, F)** MRI images.

A 55-year-old male presented with right nasal breathing difficulty and ipsilateral epistaxis for two months. He had no history of exposure to radiation, metals, wood dust or asbestos. Physical examination revealed an exophytic bleeding tumor that occupied the right nostril and nasopharynx and was implanted in the middle turbinate. A magnetic resonance imaging (MRI) and a face-neck-thorax computed tomography (CT) were performed, which revealed a 7x5 cm heterogeneous mass hyperintense on T2-weighted that occupied the entire right nasal fossa, obstructing the drainage of the paranasal sinuses and causing an erosion of the medial bony wall of the nasolacrimal duct (**Figure 1**). There was no orbital or intracranial invasion or lymph node metastasis.

**Figure 2:**
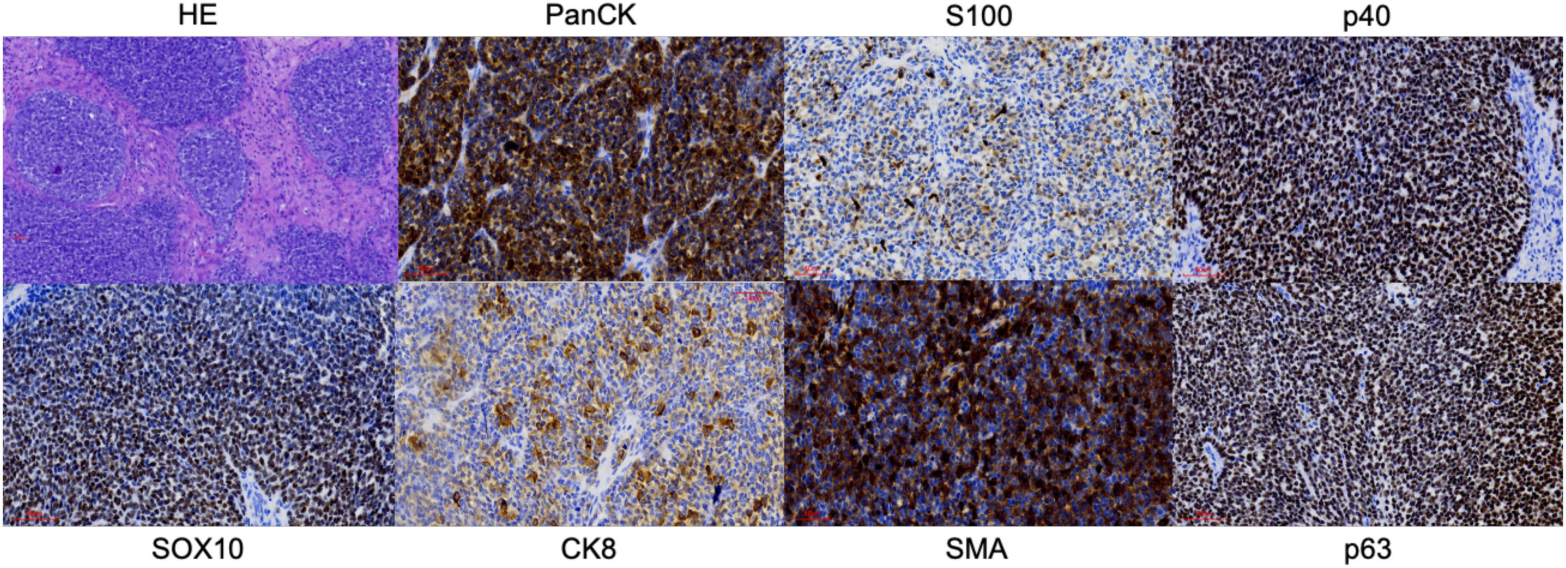
Histology (Hematoxylin and Eosin) and some of the most relevant immunohistochemical stains. Magnification x20.

**Figure 3:**
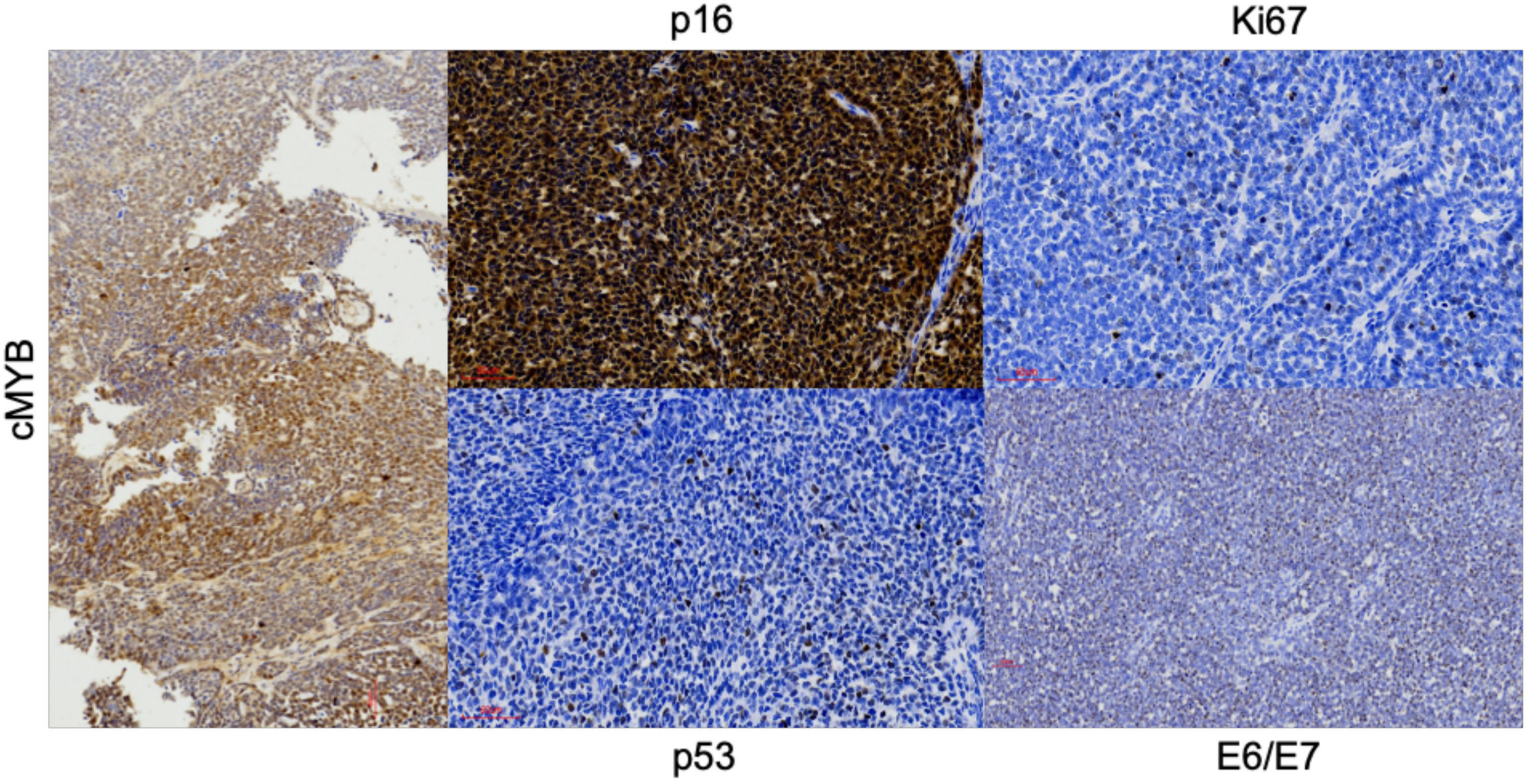
Other of the most important stains. Magnification x20.

An endoscopic biopsy of the nasal tumor was taken. Histopathological examination showed a lobulated basaloid tumor with comedonecrosis and monotonous basophilic cells that formed nests with mucinous spaces and that, in isolation, showed formation of ductal structures (**Figure 2**). Immunohistochemical study showed a predominantly myoepithelial tumor cell population with expression of S-100, SOX-10, p40, p63, SMA and Pan cytokeratin (AE1/AE3) (**Figure 2**). The ductal component showed intense positivity for CK8 (**Figure 2**). cMYB expression was moderate and variable (**Figure 3**). SMARCB1 (INI-1) showed normal expression, while NUT, Chromogranin A and Synaptophysin were negative.

Due to the diffuse p16 positivity, PCR analysis of HPV-DNA was performed and showed the presence of HPV 35. *In situ* hybridization assay for the detection of viral E6/E7 RNA was diffusely positive confirming that HPV 35 was transcriptionally active (**Figure 3**). The final diagnosis was HMSC. The results did not support the diagnosis of squamous cell carcinoma, adenoid cystic carcinoma, SMARCB1-deficient carcinoma, undifferentiated carcinoma, or NUT carcinoma. Based on these findings, an endoscopic approach with complete tumor resection (**Figure 1**) and adjuvant radiotherapy was performed. The patient is currently alive and recurrence-free after 16 months of follow-up.

**Figure 4:**
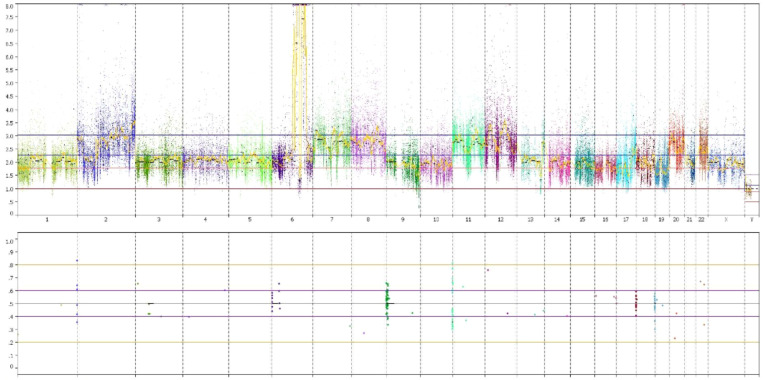
Copy number analysis: A high-level gain is observed at 6q23.

To better understand the molecular features of this tumor and due to its rarity, we performed whole exome sequencing (WES) on the tumor and matched germline DNA with a mean coverage of 126x and 122x respectively, to search for somatic mutations and copy number abnormalities. Bioinformatic analysis revealed 38 somatic sequence variants, including 30 nonsynonymous, 5 splicing, 2 frameshift, and 1 in-frame deletion mutations. Three mutated genes were indicated as likely pathogenic by ClinVar: *CHRNG, EP300* and *SUCLG1*. Four other mutated genes have been described previously in *COSMIC* and *HCGC* databases: *DIPK1C, DSCAML1, ZNF22* and *ZNF609*. In addition, the two genes carrying frameshift mutations are very likely to be considered inactivating and pathogenic: *ANKRD26* and *TBCD*. Finally, based on in-silico analyses, the following gene mutations with score 3-5 on a scale of 5 were predicted as probably pathogenic: *ARAP2, MND1, RIMS4, GRM6, DMD* and *LRIG3.* All sequencing results are given in the **Supplementary Material** (**Supplementary Table 1**). Copy number analysis deducted from the WES data showed losses at chromosomes 1p, 6q, 9q, 10, 13q, 16, 17 and 19, and gains at chromosomes 2p, 2q, 6q, 7, 8, 11, 12, 17q, 20p and 22. At 6q23 where MYB is located, a high-level gain could be observed (**Figure 4**).

We did not find mutations predictive for drug response according to the ESMO Scale for Clinical Actionability of Molecular Targets database, although targeted inhibitors of *EP300* are being evaluated in phase I/IIa clinical trials.

## Material and method

Samples of the primary tumor and peripheral blood were obtained in the Otorhinolaryngology department of the Central University Hospital of Asturias (Oviedo, Spain). Informed consent has been obtained from the patient for the study and analysis of their samples. Hematoxylin and eosin and IHC stains were performed by the Pathological Anatomy department of the Central University Hospital of Asturias in an automatic staining station (Dako Autostainer plus; DakoCytomation, Glostrup, Denmark) as part of the diagnostic routine. The following antibodies were applied (17): PanCK clone AE1/AE3, S-100 polyclonal GA504, CK8 clone DC10, Caldesmon clone h-CD, Synaptophysin clone SY38, Chromogranin A clone DAK-A3, p63 clone DAK-p63, p53 clone DO-7, Ki-67 clone MIB-1, α-SMA clone 1A4, SOX10 clone BC34 (DAKO, Glostrup, Denmark), p40 clone BC-28, p16 clone E6H4, SMARCB1 (BAF47) clone D8M1X, NUT clone C52B1 (Cell Signaling Technology, Cambridge, UK), anti-p16 clone E6H4 (Roche, Mannheim, Germany) and anti-c-Myb clone EP769Y (Abcam, Cambridge, UK). Diaminobenzidine (DAB) chromogen was used for signal detection and hematoxylin as a counterstain. Results were analyzed by the pathologist Mendoza-Pacas GE.

Tumor DNA was extracted from fresh frozen tissue using the Qiagen tissue extraction kit (Qiagen GmbH, Hilden, Germany). Germline DNA from peripheral blood was obtained using Roche high Pure Template Preparation Kit (Roche Diagnostics GmbH, Mannheim, Germany). Library preparation for whole exome sequencing was performed using the Sureselect Human All Exon V6 kit (Illumina Inc) and sequencing was performed with NovaSeq6000 150PE 18 Gb/sample (Marcogen Inc) (18). Sequencing data were processed using HD Genome One bioinformatics software (DREAMgenics, Oviedo, Spain), certified with IVD/CE marking as described previously (19). The process included quality control and alignment, somatic variant calling, variant annotation, and copy number neutral loss of heterozygosity (CN‐LOH) identification. To select somatic mutations with a pathogenic impact, we filtered out all variants with an allele frequency >5% in the normal population, all silent mutations, and all variants with a tumor frequency <10% of total reads. We considered truly relevant somatic variants to be those that appeared in the tumor sample, but not in the patient’s normal germline sample, as well as changes from a heterozygous germline variant to a homozygous tumor variant.

### HPV analysis

The presence of HPV DNA was analyzed by PCR. The quality of the extracted DNA was checked by PCR amplification of β-globin (forward primer 5’-ACACAACTTGTGTGTTCACTAGC-3’ and reverse primer 5’-CAAACTTCATCCACGTTCACC-3’). PCR with MY11/GP6+ primers (site directed L1 fragment of HPV) was performed to detect a broad spectrum of HPV genotypes (20, 21). Briefly, the PCR was performed in 25 µl of reaction mixture containing 1x PCR buffer, 2 mmol/L MgCl2, 50 µmol/L of each deoxynucleoside, 0.5 µmol/L of sense and antisense primers, 10 µl of DNA sample and 1 U Taq DNA polymerase (Promega Biotech Iberica S.L. Madrid, Spain), by thermal profile of 35 cycles: denaturation at 94°C for 30 sec, annealing at 55°C for 30 sec and extension at 72°C for 1 min, with an initial denaturation at 94°C for 5 min and a final extension at 72°C for 10 min. The amplified DNA fragments of approximately 200 bp were identified by electrophoresis in 1.5% agarose gel with ethidium bromide. All positive specimens for L1 fragment were tested by hybridization assays using type-specific probes for HPV (21).

Transcriptional activity of HPV E6/E7 genes was evaluated by RNA *in situ* hybridization (RNA ISH) (22) using the High-Risk HPV Probe set detecting HPV types of HPV types 16, 18, 26, 31, 33, 35, 39, 45, 51, 52, 53, 56, 58, 59, 66, 68, 73 and 82 (Advanced Cell Diagnostics, Hayward, CA) according to the manufacturer’s protocol. DAB chromogen was used for signal detection and hematoxylin as a counterstain. An HPV type 16-positive oropharyngeal squamous cell carcinoma (OPSCC) and an HPV-negative sinonasal squamous cell carcinoma (SNSCC) were used respectively as positive and negative controls.

CARE guidelines (2013) have been checked to write this case.

## Discussion

HMSC is provisionally included in the new classification of head and neck tumors of the WHO within non-keratinizing squamous carcinomas, requiring additional data to justify recognizing it as a single entity (3). To our knowledge and to date, 72 patients with HMSC have been reported (13, 23).

Unlike other HPV-related sinonasal tumors, HMSC is not related to smoking or occupational exposures as was the case with this patient. Radiologically, HMSC is characterized as an expansile soft tissue mass, with compressive changes of nasal structures, well-defined margins, and heterogenous enhancement without bony invasion (24). These findings were noticeable features in this case. MRI typically shows HMSC as iso to hyperintense on T2-weighted and heterogeneously enhanced on T1-weighted images after gadolinium administration (24). There is a mismatch between the clinical and radiological behavior of HMSC and its pathological aspects, as this tumor shows high-grade histological characteristics despite a clinically slow evolution and with rare metastases (2, 25, 26). The median disease-free survival is 37 months, and the local recurrence rate is low before 23 months but could exceed 40% over time (24). The different clinical evolution of HMSC compared to histologically similar tumors highlight the importance of its correct characterization. The differential diagnosis is broad and should include adenoid cystic carcinoma, basaloid squamous cell carcinoma, adenosquamous carcinoma, high-grade myoepithelial carcinoma, biphenotypic sinonasal sarcoma, NUT carcinoma, SMARCB1-deficient sinonasal carcinoma, and renal cell-like adenocarcinoma (2, 10, 13). The unique morphological and immunohistochemical features of multidirectional differentiation, including basaloid, ductal, myoepithelial, and squamous cells, overlying dysplastic change in the surface epithelium, and evidence of high-risk HPV infection, are useful in distinguishing HMSCs from other malignant tumors.

The majority of HMSC cases have a prominent solid growth pattern with minor cribriform and/or tubular patterns as in this case. The immunohistochemical study must be extensive, being essential to demonstrate myoepithelial differentiation by the tumor cells, which in the present case was confirmed by the positivity for SOX-10, SMA, p40, p63, PanCK and CK 8. Conservation of INI-1 ruled out SMARCB1-deficient sinonasal carcinoma and negativity for NUT rules out NUT carcinoma. The partial MYB expression observed in this case has been reported previously. *Andreasen S et al.* (10) found that cMYB protein expression was limited to two of six cases of HMSC, with staining in only few luminal cells whereas their four solid adenoid cystic carcinomas were diffusely positive. However, *Shan AA et al.* (11) analyzed the expression of cMYB in 10 HMSC, identifying some degree of expression of this protein in all of them, predominantly in myoepithelial cells, despite consistently lacking MYB rearrangements (1, 2, 4, 7–9). More than half showed moderate to strong intensity (11). We agree that HMSCs have a variable spectrum of MYB protein staining. Thus, regardless of scoring criteria, MYB protein has limited utility in separating adenoid cystic carcinoma from HMSC.

Diffuse positivity for p16 and detection of HPV 35 sealed the diagnosis of our case. HPV PCR or ISH testing is mandatory to confirm the diagnosis of HMSC (3, 8). The sinonasal tract is one of the so-called anatomical “hot spots” for HPV-related carcinomas since 20-30% of them are associated with this infection (14, 27, 28). Four sinonasal malignancies have been associated with HPV, including sinonasal squamous cell carcinoma, inverted papilloma-associated sinonasal squamous cell carcinoma, multiphenotypic HPV-related sinonasal carcinoma and sinonasal undifferentiated carcinoma (29). Although there is some inconsistency in the frequency and methodology used for HPV testing, most HPV-associated sinus squamous cell carcinomas are caused by HPV type 16 infection like OPSCC (14, 15). However, HMSC is most frequently associated by HPV type 33 in 67-84% of cases (2), followed by 35 in 9% of cases, as occurred in this patient. Less frequently detected types in HMSC include HPV 16 and 56 (29). All these HPV types are of the α genus, potentially oncogenic and high risk. The majority are from the same phylogenetic species, with HPV 33, 35, 16 and 52 being from the α9 species and 56 from the α6 species. The fact that they share the same species suggests common characteristics, such as tissue tropism and oncogenic potential (30).

The variability in the methodology used to detect HPV in SNSCC samples in the literature has implications for what can sensibly be inferred about the role of HPV in the formation of these tumors. For example, detection of HPV E6/E7 mRNA by reverse transcription-polymerase chain reaction (RT-PCR) or RNA ISH implies a transcriptionally active viral infection and therefore a stronger indication of a role for HPV in tumorigenesis than the presence of HPV DNA. Immunohistochemical staining for viral proteins is relatively undemanding and inexpensive, but the performance of this approach has been too inconsistent to be used as a reliable detection method (22). It is common for studies in the SNSCC literature to use p16 positivity as a surrogate marker for the presence of HPV (31, 32). p16 is an accepted indicator of HPV in the setting of OPSCC but the predictive value of p16 for HPV in SNSCC is uncertain, partly due to the low number of analyzed tumors and to the different detection methodologies. Indeed, many sinonasal carcinomas, salivary-type, and surface-type, are p16-positive in the absence of HPV (27, 33, 34).

In this case, a strong and diffuse expression of p16 was observed, HPV35 was detected by PCR and transcriptional HPV E6/E7 activity was shown by RNA ISH. Looking to the future, standardization of HPV detection using an optimal methodology is necessary to clarify the prevalence and biological relevance of HPV in sinonasal tumors and more specifically in HMSC. For this reason, we recommend performing p16, PCR and RNA ISH assays to characterize the presence of HPV.

To date, this is the first WES study to analyze mutations in HMSC. We did not find probably pathogenic mutations in the PI3K/AKT pathway previously linked to HPV-positive cancers (35) nor in the genes that encode proteins previously related to a poor prognosis in HMSC (COX-2, VEGF, EGFR, ProEx-C and TERT) (36). We also did not find mutations in *TP53*. This was expected considering the inverse association between the presence of HPV and mutations in *TP53* in other head and neck tumors (37).

Of the mutations that we defined as probably pathogenic, mutations of some of the genes encoding zinc finger proteins (ZNF), such as *ZNF750*, and the mutation in *EP300* were already described as possible candidate driver events in HPV-positive OPSCC (37, 38). In our case we found mutations in two genes that encode zinc finger proteins: *ZNF22* and *ZNF609*. ZNFs are one of the most abundant groups of proteins and have a wide range of molecular functions. Given the wide variety of zinc finger domains, ZNFs can interact with DNA, RNA, PAR (poly-ADP-ribose), and with other proteins. Therefore, ZNFs participate in the regulation of various cellular processes such as transcriptional regulation, ubiquitin-mediated protein degradation, signal transduction, actin targeting, DNA repair, cell migration, and many other processes (39).

The p300 protein, encoded by EP300, is a key member of the family of acetyltransferases and transcriptional coactivators. It is a master transcription regulator and tumor suppressor. The exact sequence variant p.Asp1399Asn is described as being located in the histone acetyltransferase domain of the protein, leading to decreased histone H3 K27 acetylation compared to the wild-type protein. p300 directly regulates the expression or activity of hundreds of genes. Many of these genes (e.g., *RB*, *TP53*, *ATM*, and *ATR*) are key factors in other signaling pathways where they control the expression or activity of many other genes. This broad influence makes p300 a crucial factor in maintaining genomic stability through DNA damage response cell cycle checkpoints, p53 regulation, and likely other mechanisms such as the Hippo pathway of which p300 is a negative regulator (40). As expected, aberrant p300 biology is linked to multiple cancers and disease states (41–44). For example, its mutation produces aberrant transcriptional silencing of genes that regulate B cell signaling and immune responses. Aberrant expression of p300 is common in hematological malignancies and is associated with chemoresistance and poor patient prognosis (45). Another example is how the destabilization of p300 by the E6 oncoprotein plays a significant role in the ability of β-HPV to cause cutaneous squamous cell carcinomas (46–49).

The role of *LRIG3* in relation to HPV neoplasms is little known. The human leucine-rich repeat immunoglobulin-like domain (LRIG) gene family includes: *LRIG1*, *LRIG2*, and *LRIG3*. *LRIG1* functions as a tumor suppressor and has demonstrated prognostic value in several human cancers, while less is known about the functions of *LRIG2* and *LRIG3*. High LRIG1 staining intensity and high fraction of LRIG3-positive cells were significantly associated with cervical adenocarcinoma patient survival, and positive correlations were found between LRIG1 and LRIG3 staining intensity and HPV status (50).

## Conclusion

HMSC is an extremely rare neoplasm with a challenging histopathologic diagnosis. It is important to consider this when dealing with a nasosinusal tumor with a solid basaloid component. This is the first report of WES analysis of an HMSC, in this case associated with the less common HPV type 35. We also identified potentially pathogenic variants, some of which have already been detected in other HPV-positive tumor types. The detected mutation in *EP300* and the overexpression of MYB may serve as molecular targets for personalized therapy.

## Data Availability

The datasets analyzed for this study can be found in Zenodo, accession number: 13270596.
