# Supplementary material for: Whole exome sequencing of human papillomavirus-related multiphenotypic sinonasal carcinoma: a case report
Source: Front Oncol. 2024 Sep 10;14:1448213. doi: 10.3389/fonc.2024.1448213 (PMC11419970; doi:10.3389/fonc.2024.1448213)
Supplement: Supplementary file 1 [file Table1.docx]

| **Gen** | **Cr** | **Protein change** | **Mutation type** | **Alle freq N** | Alle freq T | Alt reads N | Alt reads T | Coverage N | Coverage T | Cosmic ID | ICGC ID | Clin Var | DG predict |
| --- | --- | --- | --- | --- | --- | --- | --- | --- | --- | --- | --- | --- | --- |
| ABHD16B \| LOC112268269 | 20 | p.Gln321Lys \| | NO_SYN \| | 0 | 0,301703163 | 0 | 124 | 502 | 411 |  |  |  | 0,096153846 |
| ACSM2A | 16 | p.Val72Met | NO_SYN | 0 | 0,397849462 | 0 | 111 | 276 | 279 |  |  |  | 2,115384615 |
| ANKRD26 | 10 | p.Glu1512Argfs*4 | FRAMESHIFT | 0 | 0,470588235 | 0 | 16 | 17 | 34 |  |  |  |  |
| ARAP2 | 4 | p.Arg1464Pro | NO_SYN | 0 | 0,413793103 | 0 | 24 | 20 | 58 |  |  |  | 3,653846154 |
| C1QTNF12 | 1 | p.Pro42Ala | NO_SYN | 0 | 0,363636364 | 0 | 8 | 26 | 22 |  |  |  | 0 |
| CHRNG | 2 | p.Arg28Cys | NO_SYN | 0 | 0,490990991 | 0 | 109 | 167 | 222 |  |  | CONFL_INTERPR_PATHOGENICITY | 3,269230769 |
| COBLL1 | 2 | p.Cys678Phe | NO_SYN | 0 | 0,598455598 | 0 | 155 | 103 | 259 |  |  |  | 0,520833333 |
| DIPK1C | 18 | p.Cys216Tyr | NO_SYN | 0 | 0,431506849 | 0 | 63 | 178 | 146 | COSM4645311 |  |  | 3,269230769 |
| DMD | X |  | CANONICAL_SPLICING | 0 | 0,783783784 | 0 | 58 | 60 | 74 |  |  |  | 5 |
| DSCAML1 | 11 | p.Val1612Ile | NO_SYN | 0 | 0,2578125 | 0 | 33 | 109 | 128 | COSM4434213 |  |  | 0,5 |
| DSPP | 4 | p.Ser611del | INFRAME | 0 | 0,103448276 | 0 | 6 | 48 | 58 |  |  |  |  |
| EP300 | 22 | p.Asp1399Asn | NO_SYN | 0 | 0,238095238 | 0 | 15 | 59 | 63 | COSM122851 | MU1296059 | LIKELY_PATHOGENIC | 5 |
| ERCC5 \| BIVM-ERCC5 | 13 | p.Lys535Arg \| p.Lys989Arg | NO_SYN \| NO_SYN | 0 | 0,425925926 | 0 | 69 | 155 | 162 |  |  |  | 0 |
| EWSR1 | 22 | p.Pro241Gln | NO_SYN | 0 | 0,273809524 | 0 | 23 | 74 | 84 |  |  |  | 2,788461538 |
| GGCX | 2 | p.Thr739Met | NO_SYN | 0 | 0,388059701 | 0 | 26 | 106 | 67 |  |  |  | 0,384615385 |
| GRM6 | 5 | p.Pro801Ser | NO_SYN | 0 | 0,37628866 | 0 | 73 | 278 | 194 |  |  |  | 4,791666667 |
| KIAA0100 \| SPAG5-AS1 | 17 | p.Met1960Val \| | NO_SYN \| | 0 | 0,432432432 | 0 | 32 | 102 | 74 |  |  |  | 2,307692308 |
| LRIG3 | 12 |  | CANONICAL_SPLICING | 0 | 0,208333333 | 0 | 15 | 53 | 72 |  |  |  | 5 |
| MND1 | 4 | p.Trp169Leu | NO_SYN | 0 | 0,380952381 | 0 | 8 | 8 | 21 |  |  |  | 3,958333333 |
| MROH1 | 8 | p.Gly1547Arg | NO_SYN | 0 | 0,276315789 | 0 | 21 | 125 | 76 |  |  |  | 2,5 |
| OSCAR | 19 | p.Ala168Asp | NO_SYN | 0 | 0,475728155 | 0 | 49 | 174 | 103 |  |  |  | 0 |
| PCSK9 | 1 | p.Pro173His | NO_SYN | 0 | 0,5 | 0 | 59 | 121 | 118 |  |  |  | 1,25 |
| PIK3CD | 1 | p.Arg338Gly | NO_SYN | 0 | 0,42519685 | 0 | 54 | 245 | 127 |  |  |  | 1,923076923 |
| RALGAPA2 | 20 | p.Thr309Ala | NO_SYN | 0 | 0,329479769 | 0 | 57 | 74 | 173 |  |  |  | 1,634615385 |
| RIMS4 | 20 | p.Arg174His | NO_SYN | 0 | 0,245098039 | 0 | 50 | 208 | 204 |  |  |  | 4,038461538 |
| RPS27A \| CLHC1 | 2 | \| | SPLICING \| | 0 | 0,483870968 | 0 | 30 | 48 | 62 |  |  |  |  |
| SCP2 | 1 |  | SPLICING | 0 | 0,432432432 | 0 | 16 | 31 | 37 |  |  |  |  |
| SUCLG1 | 2 | p.Pro122Leu | NO_SYN | 0 | 0,459259259 | 0 | 62 | 107 | 135 |  |  | UNCERTAIN_SIGNIFICANCE | 5 |
| SYNE1 | 6 | p.Thr4621Met | NO_SYN | 0 | 0,479166667 | 0 | 69 | 126 | 144 |  |  |  | 0,192307692 |
| TBCD | 17 | p.Ala629Glnfs*31 | FRAMESHIFT | 0 | 0,177419355 | 0 | 66 | 383 | 372 |  |  |  |  |
| TG \| LOC105375768 | 8 | \| | SPLICING \| | 0 | 0,297814208 | 0 | 109 | 273 | 366 |  |  |  |  |
| TMEM30B \| PRKCH | 14 | p.Gly9Ser \| | NO_SYN \| | 0 | 0,540983607 | 0 | 33 | 51 | 61 |  |  |  | 0 |
| TTN | 2 | p.Val8039Phe | NO_SYN | 0 | 0,559322034 | 0 | 231 | 235 | 413 |  |  |  | 1,666666667 |
| UBR4 | 1 | p.Gly2951Asp | NO_SYN | 0 | 0,389312977 | 0 | 51 | 137 | 131 |  |  |  | 2,1875 |
| ZNF22 | 10 | p.Ile202Val | NO_SYN | 0 | 0,509433962 | 0 | 27 | 52 | 53 |  | MU93130564 |  | 0,384615385 |
| ZNF609 | 15 | p.Val493Ile | NO_SYN | 0 | 0,397790055 | 0 | 72 | 275 | 181 | COSM2151594 | MU591564 |  | 1,153846154 |
| ZNF646 | 16 | p.Ala1745Ser | NO_SYN | 0 | 0,459259259 | 0 | 62 | 292 | 135 |  |  |  | 0,416666667 |
| ZSCAN20 | 1 | p.Ser834Asn | NO_SYN | 0 | 0,375 | 0 | 33 | 125 | 88 |  |  |  | 0,568181818 |
